# HIV infection and exposure is associated with increased cariogenic taxa, reduced taxonomic turnover, and homogenized spatial differentiation for the supragingival microbiome

**DOI:** 10.1186/s40168-025-02123-9

**Published:** 2025-06-16

**Authors:** Allison E. Mann, Ciara Aumend, Suzanne Crull, Lauren M. O’Connell, Esosa Osagie, Paul Akhigbe, Ozoemene Obuekwe, Augustine Omoigberale, Matthew Rowe, Thomas Blouin, Ashlyn Soule, Colton Kelly, the DOMHaIN Study Team, Oghenenero Igedegbe, Ruxton Adebiyi, Matron Christy Ndekwu, Uwagboe Odigie, Oyemwen Olaye, Ehioze Awanlemhen, Samuel Chukwumaeze, Matthew Imoe, Daniel Oakhu, Susan Dare, Nosakhare Idemudia, Osasumwen Ehigie, Kelly Avenbuan, Amara Godwins, Nneka Chukwumah, Stanley Iyorzor, Owen Omorogbe, Chioma Ugorji, Robert A. Burne, Modupe O. Coker, Vincent P. Richards

**Affiliations:** 1 Department of Anthropology, University of Wyoming, Laramie, WY 82071 USA; 2 Department of Biological Sciences, Clemson University, Clemson, SC 29634 USA; 3 Institute of Human Virology Nigeria, Abuja, Nigeria; 4 University of Benin Teaching Hospital, Benin, Edo State, Nigeria; 5 Department of Oral Biology, College of Dentistry, University of Florida, Gainesville, FL USA; 6 Department of Basic and Translational Sciences, Penn Dental Medicine, University of Pennsylvania, Philadelphia, PA 19104 USA; 7 Center for Clinical and Translational Research, Penn Dental Medicine, University of Pennsylvania, Philadelphia, USA; 8 Department of Oral Biology, Rutgers School of Dental Medicine, Rutgers University, Newark, USA

**Keywords:** Oral microbiome, HIV, Longitudinal analysis, Caries disease, CD4 count

## Abstract

**Background:**

The oral microbiome consists of distinct microbial communities that colonize various ecological niches within the oral cavity, the composition of which are influenced by nutrient and substrate availability, host genetics, diet, behavior, age, and other diverse host and environmental factors. Unlike other densely populated human-associated microbial ecosystems (e.g., gut, urogenital), the oral microbiome is directly and frequently exposed to external influences, contributing to its relatively lower stability over time. In individuals with compromised immunity, such as those living with HIV, the composition and stability of the oral microbiome may be especially vulnerable to disruption. Cross-sectional studies of the oral microbiome in children living with HIV capture a glimpse of this temporal dynamism, yet a full appreciation of the relative stability, robusticity, and spatial structure of the oral environment is necessary to understand the role of microbial communities in promoting health or disease in the context of HIV. Here, we investigate the spatial and temporal stability of the oral microbiome over three sampling time points in the context of HIV infection and exposure. Individual teeth were sampled from a cohort of 565 Nigerian children with varying levels of tooth decay severity (i.e., caries disease). We collected 1960 supragingival plaque samples and characterized the oral microbiome using a metataxonomic approach targeting an approximately 478 bp region of the bacterial *rpo*C gene.

**Results:**

Both HIV infection and exposure have significant, if subtle, effects on the stability of the supragingival plaque microbiome. Specifically, we observed (1) a slight but significant reduction in taxonomic turnover among HIV-exposed and infected children; (2) an association between HIV infection and a more homogenized oral community across the anterior and posterior dentition in children living with HIV; and (3) a relationship between impaired immunity, lower taxonomic turnover over time, and an elevated frequency of cariogenic taxa, including *Streptococcus mutans*, in children living with HIV.

**Conclusions:**

Despite the influence of various contributing factors, we observe an effect of HIV status on both the temporal and spatial stability of the oral microbiome. Specifically, the results presented here indicate that the oral microbiome shows less community change over time in children living with or exposed to HIV, which we hypothesize may be linked to a reduced capacity to adapt to environmental changes. The observed taxonomic rigidity among children living with HIV may signal community dysfunction, potentially leading to a higher incidence of oral diseases, including caries, in this cohort.

Video Abstract

**Supplementary Information:**

The online version contains supplementary material available at 10.1186/s40168-025-02123-9.

## Background

The human oral microbiome is a complex and dynamic ecosystem, comprised of diverse microbial communities that inhabit distinct ecological niches across the oral cavity [1–6]. The most densely populated of these ecological niches are microbial biofilms that form highly structured communities on the teeth (i.e., dental plaques) [7, 8], the composition of which is further shaped by variation in substrate availability, salivary flow, and the presence of other co-aggregating taxa [2, 9–11]. In addition to host genetics, the composition of the oral microbiome is influenced by diet (primarily the intake of fermentable carbohydrates), behavior, and age [12–18], and is particularly susceptible to disruptions due to changing environmental conditions [15, 19, 20]. For individuals with compromised immunity, such as those living with HIV, these disruptions may be amplified, affecting the stability and health of the microbiome in ways that may increase vulnerability to conditions like dental caries.

Highly active antiretroviral treatment (HAART) has substantially decreased mortality and improved the quality of life of children living with HIV. However, despite these advances, children living with HIV continue to face unique health challenges, particularly regarding oral health. In addition to an increased prevalence of oral candidiasis and periodontal disease [21–24], children living with HIV have a higher prevalence and more severe clinical presentation of dental caries in both primary and permanent dentition, the impact of which is associated with viral load and individual immune status [25–30]. Previous work by our group found a small but distinct effect of HIV status on the supragingival plaque microbiome which is exacerbated in the context of severe caries [31]. Moreover, there is growing evidence that perinatal exposure to HIV has systemic effects on the health, growth, and development of children [32–38] and recent research by our group found a distinct oral mycobiome [39], decreased salivary flow [40], increased developmental defects of the enamel surface [41], and increased incidence of stunting in both children exposed to and infected by HIV (manuscript in preparation).

Given the relative susceptibility of the oral microbiome to perturbations as compared to other microbe-rich host-associated environments, cross-sectional studies only provide a brief snapshot of the ecological diversity and dynamics of the oral microbiome over time, and the impact of illness or stress on the long-term stability of the oral community is poorly defined. In the current study, we compare the temporal and spatial stability of the oral microbiome among children living with HIV currently on HAART (HI), children perinatally exposed to but uninfected by the virus who were treated with nevirapine at birth and during breastfeeding (HEU), and unexposed and therefore uninfected children (HUU).

In this study, we aimed to assess the impact of HIV infection and exposure on the stability and diversity of the oral microbiome with a particular focus on the development of caries in children living with and exposed to HIV. Notably, as the children living with HIV in this cohort are receiving HAART, we are unable to isolate the effects of HIV alone from those of the treatment itself or from a possible combined influence of both HIV and HAART on the oral microbiome. Despite these and other complexities, we detect a slight but significant impact of HIV infection and exposure on the temporal and spatial stability of the oral microbiome. To explore this, we conducted a longitudinal collection of supragingival plaque samples across three distinct time points using a metataxonomic approach targeting a fragment of the bacterial *rpo*C gene. Using these data, we examined the degree of taxonomic turnover within HIV status groups, spatial differentiation of the microbial communities inhabiting anterior and posterior teeth, and the association between HIV status and caries-related taxa. While we detect significant variation across individuals independent of HIV status, this study provides insights into how HIV infection and exposure impact microbial community dynamics over time and highlights the potential for microbial stability to contribute to caries disease, particularly in immune-compromised children. Specifically, this research indicates that children living with and exposed to HIV experience lower taxonomic turnover over time, that children living with HIV have altered spatial distribution of microbial communities across their dentition, and that low taxonomic turnover is linked to a higher abundance of taxa associated with caries disease. Understanding the relative stability of microbial communities over time is critical for improving oral health outcomes in this population, as well as informing targeted prevention and intervention strategies.

## Methods

### Study design and sample collection

Samples included in this study were collected from 565 children between May 2019 and February 2021 during three separate clinical visits at the University of Benin Teaching Hospital in Benin City, Nigeria. In total, we collected 1960 supragingival plaque samples as part of the Dental Caries and its Association with Oral Microbiome and HIV in Young Children – Nigeria (DOMHaIN) Study [28]. A total of 615 supragingival plaque samples were collected from uninfected and unexposed children (HUU), 614 from children exposed to HIV in utero but uninfected with the virus (HEU), and 731 from children living with HIV (HI). A summary table of study participants and sample demographics can be found in Table 1.

**Table 1:** Demographic summary and count of study participants and plaque samples

		Number of plaque samples				Number of individuals			
Variable	Category	Visit 1	Visit 2	Visit 3	Total	Visit 1	Visit 2	Visit 3	Total
Sex	Female	342	272	286	900	223	272	286	781
	Male	404	324	332	1060	259	324	332	915
HIV status	HEU	225	189	200	614	161	167	169	497
	HI	289	217	225	731	167	158	162	487
	HUU	232	190	193	615	154	153	159	466
Caries experience	D	102	70	69	241	71	70	69	210
	E	69	55	58	182	55	55	58	168
	H	575	471	491	1537	468	471	491	1430
Age (years)	3	32	5	5	42	23	5	5	33
	4	74	59	63	196	52	59	63	174
	5	92	77	81	250	63	77	81	221
	6	99	60	65	224	61	60	65	186
	7	140	113	111	364	94	113	111	318
	8	126	101	112	339	68	101	112	281
	9	129	86	84	299	92	86	84	262
	10	54	91	91	236	30	91	91	212
	11	0	4	6	10	0	4	6	10

Supragingival plaque samples were collected from a single tooth and categorized into one of six progressive health categories (Table 2). Patients were asked to abstain from eating for at least 2 h and from performing any oral hygiene, such as brushing or flossing the teeth, for 12 h prior to sample collection. In brief, we collected each plaque using a sterile curette which was then stored in a sterile 2-mL cryogenic vial containing 500 μL of RNAlater. Post-collection, samples were placed immediately on ice and stored at − 80 °C within 2 h of collection. Detailed sampling procedures are described in Coker et al. [28]. Teeth were selected for sampling using a standardized method based on oral health assessments conducted by a trained dentist. At each visit, overall and individual tooth health were evaluated using the International Caries Detection and Assessment System (ICDAS) [42]. For each study participant, the tooth with the highest disease score was selected for sampling. If multiple teeth had the same disease status, molars and premolars were prioritized. When disease status was consistent across the full dentition (e.g., classified as H-CF), a tooth was chosen randomly for sampling. For diseased teeth (i.e., E or D), plaque was collected directly from the lesion.

**Table 2:** Nested tooth and oral health classification scheme

Individual tooth health	Participant oral health	Individual tooth health + participant oral health
Healthy (H)	Caries free (CF)	Health tooth from caries-free mouth (H-CF)
	Enamel caries present (CE)	Healthy tooth from a mouth with active enamel caries (H-CE)
	Dentin caries present (CD)	Healthy tooth from a mouth with active dentin caries (H-CD)
Enamel cavity present (E)	Enamel caries present (CE)	Tooth with an enamel lesion from mouth with active enamel caries only (E-CE)
	Dentin caries present (CD)	Tooth with an enamel lesion from a mouth with active dentin caries (E-CD)
Dentin cavity present (D)	Dentin caries present (CD)	Tooth with a dentin cavity from a mouth with active dentin cavities (D-CD)

The same teeth were not specifically targeted at each visit; instead, the selection process was repeated as outlined above. However, a subset of the same teeth collected from the same individual over two or more time points were used for taxonomic turnover analyses described below. On average, the intervals between sample collections were as follows: between visits one and two, 182 ± 28 days; between visits two and three, 222 ± 58 days; and between visits one and three, 401 ± 81 days. Only teeth with matching World Dental Federation (FDI) codes [43] across visits were included in matched plaque analyses, excluding comparisons between deciduous and permanent teeth. Table 3 presents the number of unique and total plaque samples collected at each time point.

**Table 3:** Counts of teeth sampled at each time point excluding teeth where FDI not known

	Visit 1	Visit 2	Visit 3	New samples
Visit 1	699	-	-	699
Visit 2	184	586	-	402
Visit 3	204	255	608	149

### DNA extraction, library preparation, and sequencing

We extracted DNA from each sample using the DNeasy PowerBiofilm kit (Qiagen, Valencia, CA, USA) following the manufacturer’s suggested protocol. We quantified the total DNA yield post-extraction for each sample using a Qubit fluorometer (Invitrogen, Carlsbad, CA). To track potential sources of contamination, an extraction blank using molecular-grade water was processed in parallel to all samples. To characterize the bacterial community, we amplified a fragment of the bacterial *rpo*C gene using custom primers (rpoCF: 5′ – MAYGARAARMGNATGYTNCARGA – 3′; rpoCR: 5′ – GMCATYTGRTCNCCRTCRAA – 3′) as described in Mann et al. [31]. Each PCR reaction consisted of the following: 0.5 μL each of the forward and reverse primers, 10 μL molecular grade water, 4 μL of template DNA, and 10 μL of the Platinum Hot Start PCR Master Mix (Invitrogen, Carlsbad, CA, USA). We adopted this approach instead of the more commonly used 16S rRNA metabarcoding methods as previous research has shown that the 16S rRNA locus has poor resolution for common oral groups (e.g., the streptococci) [44], suffers from recombination, a poor overall phylogenetic signal, and exists as multiple copies within a genome [45, 46]. By contrast, the *rpo*C gene is single copy, has a strong phylogenetic signal, and does not show evidence for recombination [45] and thus likely better recapitulates ecological diversity of the supragingival plaque microbiome as compared to 16S rRNA metabarcoding.

We processed PCR blanks (molecular grade water) in parallel to all samples to track sources of contamination. Each reaction was amplified using the following thermocycler conditions: 94 °C for 3 min followed by 41 cycles of 94 °C for 45 s, 39.5 °C for 1 min, and 72 °C for 1 min 30 s. A final elongation step was performed for 10 min at 72 °C. Amplification of all samples was confirmed through both gel electrophoresis and Qubit fluorometer (Invitrogen, Carlsbad, CA, USA). Finally, we pooled each sample library at equimolar concentrations and sequenced the final pools on an Illumina MiSeq using V3 2 × 300 paired-end chemistry (Illumina, San Diego, CA, USA). One microliter of each PCR blank was added to the pool and sequenced in parallel to all samples to track potential sources of contamination.

### Computational analyses

We first removed primers and adapter sequences from our raw sequencing read files using Cutadapt (v 1.18) [47]. Next, we quality filtered, merged, generated Amplicon Sequence Variants (ASVs), and removed suspected chimeric sequences using DADA2 (v 1.22.0) [48] in an R (v 4.1.0) environment [49]. Quality-filtered paired-end reads that were shorter than 450 bp after merging were removed from downstream analysis. We next assigned a taxonomy to each ASV using Kraken2 (v 2.1.2) [50] and a custom *rpo*C database as reference [31]. ASVs that could not be assigned to the phylum level or below (i.e., kingdom only) were removed from analysis. We removed low-abundance ASVs with a prevalence threshold of 0.1% (*n* = ~ 2 samples) unless they had a total abundance of more than 1000 reads, in which case the ASV was retained for downstream analysis. Finally, we removed samples with fewer than 5000 reads post-filtering. Rarefaction curves for all samples post-quality filtering can be found in Figure S1.

We next performed diversity analyses for the filtered dataset using the R libraries phyloseq (v 1.38.0) [51], vegan (v 2.6–4) [52], compositions (v 2.0–6) [53], and microbiome (v 1.16.0) [54]. Significance in diversity metrics between groups was determined using PERMANOVA analysis (vegan::adonis2) performed on center log-ratio (CLR) transformed data. We used coda4 microbiome (v 0.1.4) [55] to identify microbial signatures that are predictive of sample metadata. This method calculates the minimum number of features (here, bacterial species) that have the maximum predictive power for a particular user-defined metadata category. A microbial signature, therefore, is defined by the relative abundance of two groups of taxa wherein the balance of taxa with positive and negative coefficients has the highest correlation with your chosen variable. Finally, we used the R package effectsize [56] to assess the strength of statistical associations.

We calculated the taxonomic turnover of the oral microbiome on a single tooth over time following the protocol detailed in Bastiaanssen et al. [57]. In brief, we defined taxonomic turnover as the absolute Euclidean distance between paired plaque samples from the same tooth and individual over two or more collection time points using an Aitchison distance matrix calculated from CLR transformed count data.

Next, we wanted to identify conserved microbial associations over time (i.e., the co-occurrence of species pairs on distinct teeth/samples) within and between our HIV status and tooth health groups. To do this, we first generated microbial association networks with NetCoMi (v 1.1.0) [58] and Spiec-Easi (v 1.1.3) [59] using the neighborhood selection method (MB) [60]. Before network generation, we collapsed our ASV frequency table to the species level and only included species found in at least 1% of all samples with a minimum of 10 observations to minimize the effect of low frequency taxa. We generated microbial association networks for each HIV status (HI, HEU, HUU) and tooth health group (H, E, D) at each sampling time point (three networks per group) and used anuran (v 1.1.0) [61] to depict a single Core Association Network (CAN) for each HIV status or tooth health group across all time points (set size = 1.0). Anuran identifies conserved patterns (i.e., groupings) across networks. As such, CANs generated for this study represent the consensus of microbial associations over time within our groups. In addition, we generated a “global” CAN using all samples to act as a baseline comparison to our group-specific CANs (set size = 0.6). Next, we used the greedy clustering algorithm implemented in igraph (v 1.6.0) [62] to cluster species and quantify cluster modularity within each CAN [63]. Only clusters with at least ten or more members were analyzed.

Finally, we wanted to better understand the community ecology on individual teeth directly before or after the tooth has a high relative abundance of the cariogenic taxon *S. mutans*. While *S. mutans* is a dominant taxon in many supragingival plaque samples collected from late-stage cavitated teeth, it is often absent or at very low frequency at the early stages of tooth decay (i.e., enamel lesions or white spots) [31, 64, 65]. As such, it is unclear what role (if any) *S. mutans* or other bacterial groups play in the initiation of caries. Conversely, many late-stage cavitated teeth have very little or no *S. mutans* which may suggest that the proliferation of *S. mutans* during the intensification of tooth decay is self-limiting and the community collapses over time [31]. As such, it is important to know if there are predictable taxa that recolonize the tooth and if this recolonization recapitulates the original plaque community.

To better understand the plaque environment before or after the proliferation of *S. mutans*, we first identified all individual teeth that were sampled from the same child at more than one time point. From this, we identified teeth that had a relatively low proportion of *S. mutans* (≤ 5%) either before or after the same tooth had a relatively high proportion of *S. mutans* (≥ 10%). Using this subset of paired samples of the same tooth over time, we first performed a random forest classification analysis using ranger (v 0.16.0) [66] to determine if the microbial community can be used to predict later *S. mutans* colonization and proliferation (i.e., before *S. mutans*) or if the community that recolonizes the tooth after *S. mutans* collapse is consistent and predictable (i.e., after *S. mutans*). Finally, we used a post hoc analysis of our random forest classification model to identify specific bacterial species that are explanatory of our two groups (either before or after *S. mutans*) using FastShap (v 0.1.1) [67].

Conda environments and all scripts for data analysis can be found at https://github.com/aemann01/long_oral_microbiome and are archived at Zenodo (10.5281/zenodo.11396311) for analytical reproducibility.

## Results

### Sample demographic and taxonomic summary

After quality filtering and removal of samples with low read counts, we retained 14,111 unique ASVs assigned to a total of 1960 individual supragingival plaque samples collected from 565 children. Our final sample demographic includes 900 samples collected from female participants and 1060 from male participants. Of the plaque samples, 746 were collected at visit one, 596 at visit two, and 618 at visit three. The average age of participants at visit one was 6.9 years old (SD ± 1.9) and 7.2 years old (SD ± 2.0) at visit three (Figure S2). Of all plaque samples retained post-quality filtering, 38% originated from an HI participant, 31% from a HEU participant, and 31% from a HUU participant. Full sample metadata can be found in Table S1.

The top phyla found across all samples and all visits included *Bacteroidetes* (average proportion 31%), followed by *Firmicutes* (26%), *Proteobacteria* (21%), *Actinobacteria* (16%), and *Fusobacteria* (15%) (Table S2; Figure S3). Top genera include *Streptococcus* (66%), *Ligilactobacillus* (64%), *Rothia* (56%), *Capnocytophaga* (55%), and *Prevotella* (55%) (Table S2). HEU samples across all three visits had significantly higher alpha diversity as measured by the observed number of ASVs as compared to HI samples (*p* = 0.017) but not compared to HUU samples, and D-CD samples had significantly lower alpha diversity as measured by Shannon diversity as compared to E-CD and any healthy tooth independent of the overall oral health (*p* < 0.0001) (Figure S4).

### Children unexposed and uninfected with HIV have a higher rate of taxonomic turnover over time as compared to other children in this cohort

We defined taxonomic turnover on the same tooth as the absolute Euclidean distance between paired plaque samples using an Aitchison distance matrix where high taxonomic turnover indicates greater dissimilarity over time, while low taxonomic turnover indicates greater similarity. In total, 184 teeth were sampled at both visit one and visit two, with an average of 182 days between visits; 255 teeth were sampled at both visit two and visit three, with an average of 222 days between visits; and 203 teeth were sampled at both visit one and visit three, with an average of 403 days between visits.

**Fig. 1:**
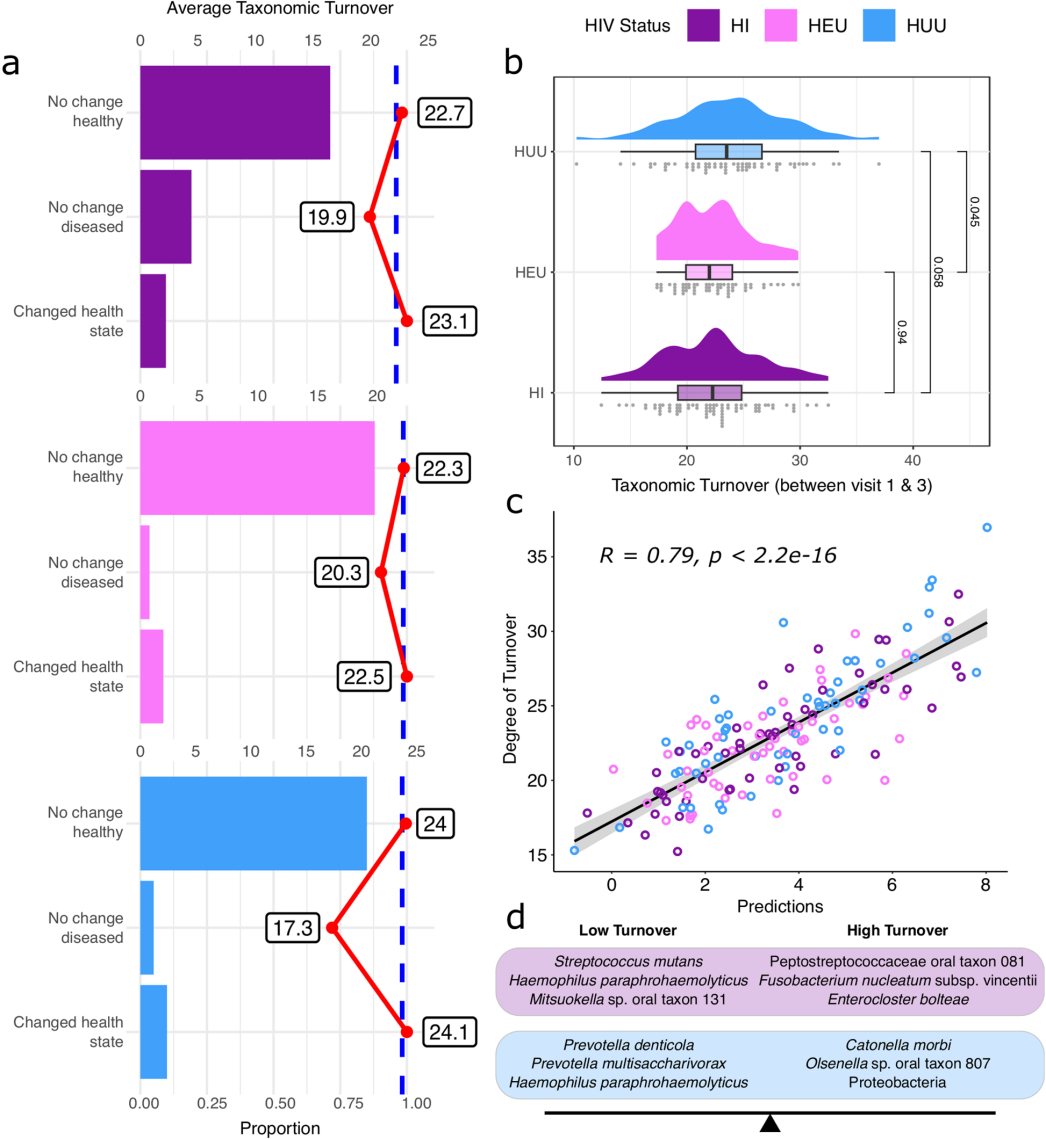
Taxonomic turnover on individual teeth is higher in HUU as compared to HEU and HI children. (a) Histograms of the proportion and average taxonomic turnover for teeth collected at visit one and visit three that had (top) no change in health status and remained healthy, (middle) no change in health status and remained in a diseased category (i.e., E or D), or (bottom) changed in health status (either towards a more healthy or more diseased category). Blue dotted lines represent the global average taxonomic turnover for all samples within an HIV status group. Red lines and corresponding number represent the average taxonomic turnover for that particular health change category. (b) Distribution of taxonomic turnover scores for each of the three groups at visit one versus visit three. (c) Predictions plot of the microbial signature model generated using all teeth with more than one sampling point (visit one, two, and three). Correlation coefficient and *p* value calculated from all samples in the plot independent of HIV status. (d) The top three weighted taxa in the microbial signature model for high or low taxonomic turnover for HI children (purple) and HUU children (blue). There were no significant taxa detected for HEU children

Overall, we observed a high degree of taxonomic turnover across all teeth and individuals, with no significant differences between HIV status groups when comparing visit one to visit two or visit two to visit three. However, a slight but significant difference was detected when comparing visit one and visit three. When analyzing all teeth regardless of health status, we found a small but significantly higher degree of taxonomic turnover in HUU as compared to HEU children (*p* = 0.045; Cohen’s *d* = 5.86e-03 ± − 0.34, 0.35) and a small but moderately significantly (i.e., a *p* value between 0.05 and 0.1) higher degree as compared to HI children (*p* = 0.058; Cohen’s *d* = − 0.33 ± − 0.66, 0.01) which varies according to the health of each tooth between sampling points (Fig. 1a, 1b). Similarly, when comparing healthy teeth only (H), we found a small but significantly higher degree of turnover in HUU compared to HEU children (*p* = 0.049; Cohen’s *d* = − 0.45 ± − 0.83, − 0.06), but no significant difference between HUU and HI children (*p* = 0.154; Cohen’s *d* = − 0.30 ± − 0.68) or HI and HEU children (*p* = 0.67; Cohen’s *d* = − 0.13 ± − 0.51). While these patterns are subtle and there are likely many other influential factors, these findings hint at underlying dynamics in the oral microbiome that vary between HIV status groups and thus warrant further investigation, particularly over longer time periods. We found no significant differences in taxonomic turnover when comparing male and female patients (*p* = 0.63), by CD4 group (low, normal, or high) (Kruskal Wallis, *p* = 0.5) or when comparing the largest group of older children (10 years) to the largest group of younger children (4 years) (*p* = 0.63). We did not compare the youngest (3 year olds, *n* = 7) to the oldest children (11 year olds, *n* = 1) due to small sample sizes.

We next identified microbial signatures of high or low taxonomic turnover across any tooth with two or more sampling time points. The resulting microbial signature is defined by the relative abundance of two groups of taxa where taxa with negative coefficients are correlated with low taxonomic turnover and those with positive coefficients are correlated with high taxonomic turnover. The absolute value of the coefficient is reflective of the degree of impact of that taxon on the model. From this analysis, we found a positive linear association between the degree of taxonomic turnover and the resulting microbial signature prediction (*R* = 0.79, *p* < 2.2e-16) (Fig. 1c). Among samples from children living with HIV, taxa associated with low taxonomic turnover include *S. mutans* (coeff − 0.42), *Haemophilus paraphrohaemolyticus* (coeff − 0.21), *Mitsuokella* sp. oral taxon 131 (coeff − 0.15), *Prevotella multisaccharivorax* (coeff − 0.14), and *Neisseria cinerea* (coeff − 0.08) (Fig. 1d). Taxa associated with high taxonomic turnover in children living with HIV include *Peptostreptococcaceae* bacterium oral taxon 081 (coeff 0.29) followed by *Fusobacterium nucleatum* subsp. vincentii (coeff 0.24), *Enterocloster bolteae* (coeff 0.21), *Prevotella intermedia* (coeff 0.16), *Leptotrichia buccalis* (coeff 0.08), and an uncharacterized *Treponema* species (coeff 0.02) (*R* = 0.8, *p* = 3.9e-13). While our initial analysis classified this taxon as *Treponema phagedenis*, this species is not associated with the oral cavity [68]. Further analysis using BLAST found that this ASV has the highest pairwise identity with *T. vincentii* (98% coverage, 81.32% ID) followed by *T.* sp. OMZ (98% coverage, 81.0% ID) and *T. phagedenis* (99% coverage, 80.88% ID) and therefore likely represents a closely related strain of oral-associated *Treponema* that was misclassified as *T. phagedenis*. An additional analysis placing this ASV in a tree of full-length *Treponema rpo*C genes (Figure S5) further supports this assumption as the ASV generated in this study falls sister to *T. vincentii* and *T. medium*, both of which are known residents of the oral cavity [69]. Finally, among HUU children, *Prevotella denticola* (coeff − 0.5), *P. multisaccharivorax* (coeff − 0.43), and *H. paraphrohaemolyticus* (coeff − 0.07) are associated with low taxonomic turnover while *Catonella morbi* (coeff 0.6), *Olsenella* sp. oral taxon 807 (coeff 0.15), an unknown species of Proteobacteria (coeff 0.15), and *Solobacterium moorei* (coeff 0.09) are associated with high taxonomic turnover (Fig. 1d; Table S4). We detected no microbial signature of taxonomic turnover among HEU children. While we found no significant differences in the degree of taxonomic turnover among older and younger children in this cohort, we detected a significant association of specific marker taxa and increasing age (*R* = 0.43, *p* < 2.2e-16). Specifically, we found *Prevotella* sp. oral taxon 317 (coeff 0.4), *Treponema* sp. (previously misclassified as *T. phagedenis* coeff 0.33), *Leptotrichia* sp. oral taxon 417 (coeff 0.15), and *Porphyromonas gingivalis* (coeff 0.12) to be associated with older children. Conversely, *Neisseria* sp. oral taxon 014 (coeff − 0.21), *Leptotrichia goodfellowii* (coeff − 0.19), *Campylobacter curvus* (coeff − 0.17), *Blautia* sp. LZLJ-3 (coeff − 0.15), *Haemophilus* sp. HMSC073 C03 (coeff − 0.11), *Eubacterium yurii* subsp. margaretiae (coeff − 0.09), *Rhizobiales* bacterium IZ6 (coeff − 0.06), and *Capnocytophaga* sp. oral taxon 863 (coeff − 0.02) with younger children (Figure S6). Similarly, while we did not detect a significant difference between the taxonomic turnover among males and females, we found an unidentified *Proteobacteria* to be more highly associated with males (coeff 1) while *Campylobacter gracilis* (coeff − 0.54) and *Dialister invisus* (coeff − 0.46) was more highly associated with females (Figure S7).

### Despite differences in taxonomic turnover on individual teeth, across all samples, there are groups of species that are consistently co-associated over time

**Fig. 2:**
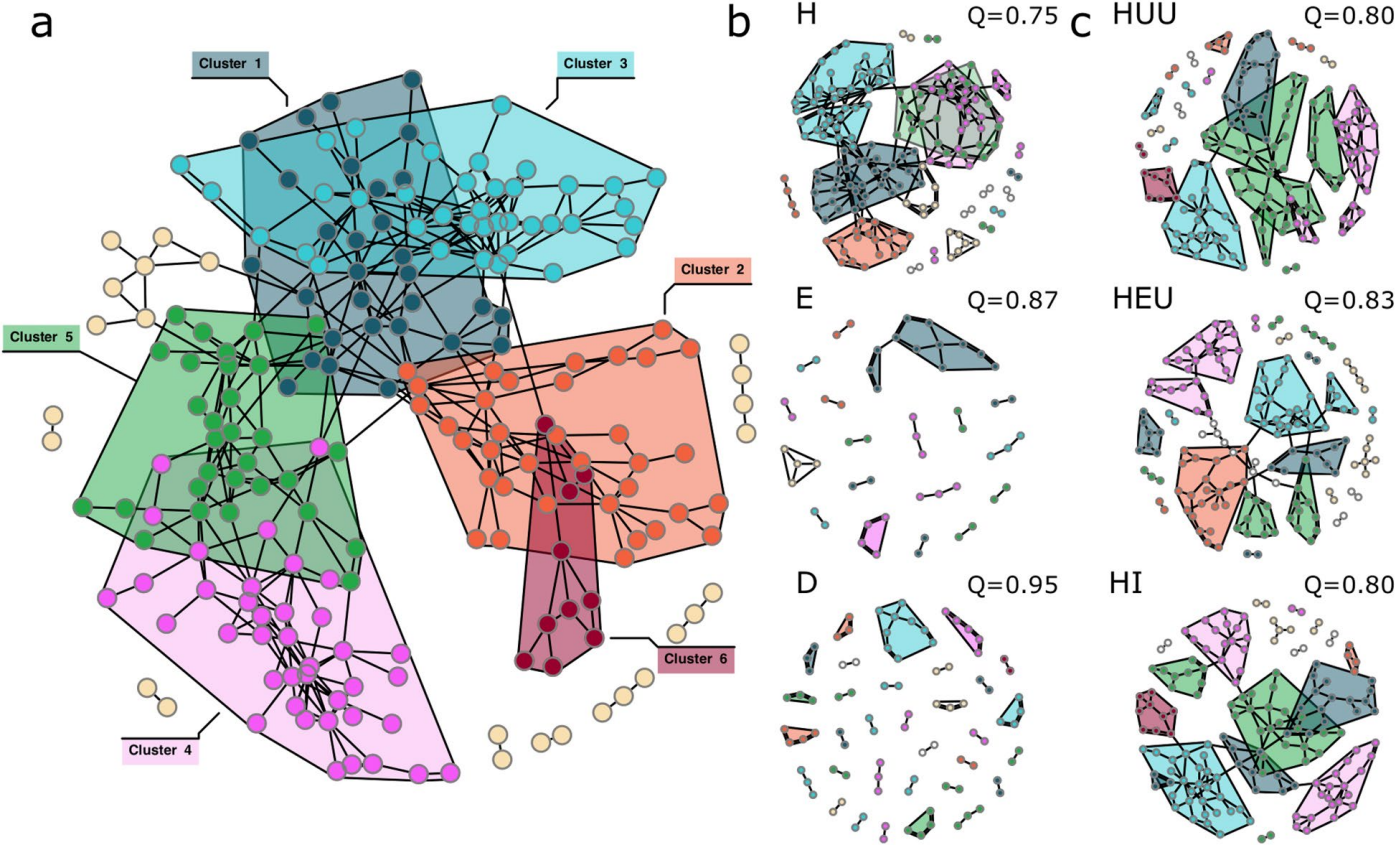
High modularity of core association networks (CANs) associated with early and late-stage caries disease. (a) Global CAN generated by comparing all samples across all three visits. We identified six clusters of co-associated taxa with variable predicted functional outcomes. Cluster six includes taxa commonly associated with caries disease (red). Clusters defined by this global CAN network are used to color code HIV status group and individual tooth health networks (b and c). (b) CANs by individual tooth health. Healthy teeth (H) have a substantially more interconnected network with low modularity (Q). Both teeth with enamel lesions (E) and dentin cavities (D) have extremely sparse and poorly connected networks with high modularity. (c) CANs across all three visits grouped by HIV status. Modularity of each network denoted for each CAN (Q). Polygons grouping clusters on each network are specific to that HIV status group CAN. Colors represent cluster identity from global CAN (a). White nodes are nodes unique to that CAN

We next used core microbial association networks (CANs) to identify clusters of species that are consistently co-associated with one another on teeth over all three clinical visits. First, we created a “global” CAN generated from all plaque samples across all three visits to act as a baseline comparison to group-specific CANs. In our global CANs, we detected six distinct cluster communities, the largest of which were cluster 2 (*n* = 35), cluster 3 (*n* = 38), and cluster 4 (*n* = 39) and the smallest, cluster 6 (*n* = 10) (Fig. 2a; Table S5). In general, co-associated species within clusters appear to have similar functional or clinical relevance. For example, commensal and species important for biofilm formation and structure dominate cluster 4 (e.g., *Streptococcus sanguinis*,* Streptococcus gordonii*, *Neisseria mucosa*, *Haemophilus parainfluenzae*,* Streptococcus oralis*,* Streptococcus mitis*, and *Corynebacterium durum*) and cluster 5 (e.g., *Leptotrichia* sp. oral taxon 215, *Leptotrichia* sp. oral taxon 212, *Corynebacterium matruchotii*,* Streptococcus cristatus*) [3, 10, 70] while clusters 1, 2, and 3 include a mixture of suspected commensal and potential pathogenic species. For example, cluster 2 includes a variety of periodontal pathogens including members of the classic “red complex” in the etiology of periodontal disease (i.e., *Treponema denticola*, *Tannerella forsythia*, and *Porphyromonas gingivalis*) as well as species that previously have been isolated from periodontal pockets or coaggregate with other periodontal pathogens including *Eubacterium nodatum*, *Eubacterium saphenum*, *Filifactor alocis*, *Porphyromonas endodontalis*, and *Treponema medium* [71–76]. The smallest cluster, cluster 6, includes species almost exclusively associated with caries disease including *S. mutans*, *Scardovia wiggsiae*, *Propionibacterium acidifaciens*,* P. multisaccharivorax*,* P. denticola*, and *Scardovia inopinata* [65, 77–84].

Next, to better understand how these core association networks differ across tooth health and HIV status groups, we calculated community modularity (Q) across all three visits within individual tooth health and HIV status groups. Modularity is a quantitative measure of network community structure wherein networks with high community modularity have more distinct (but potentially smaller) clusters that are themselves densely connected to other members of that cluster and at the same time are only loosely connected (or disconnected) from other clusters [85]. Conversely, low community modularity is reflective of fewer distinct, but potentially larger clusters of densely connected taxa. As our networks represent a consensus of co-associated taxa across all three sample visits, we expect that low community modularity (i.e., fewer distinct cluster groups) reflects higher core taxonomic stability over time.

We find that community modularity among all healthy teeth (H) is relatively low (Q = 0.75) and increases as the disease progresses to enamel lesions (Q = 0.87) and eventually to dentin lesions (Q = 0.95) (Fig. 2b). Within HIV groups, modularity of both our HUU and HI CANs were equivalent at Q = 0.80 while our HEU CAN had slightly higher modularity at Q = 0.83 (Fig. 2c). This suggests that while the bacterial community inhabiting individual teeth among HEU children changes little over time (i.e., low turnover), the community is less cohesive and more fragmented. Moreover, cluster 6 is completely absent from the HEU CAN and conversely is the only one of the three HIV status groups to have a substantial cluster representative of global cluster 2, potentially indicative of differences in susceptibility to caries vs periodontal disease.

High levels of *S. mutans* on a single tooth are preceded by the presence of taxa typically associated with oral health, and the community fails to revert to its original composition after the collapse of the *S. mutans* population.

**Fig. 3:**
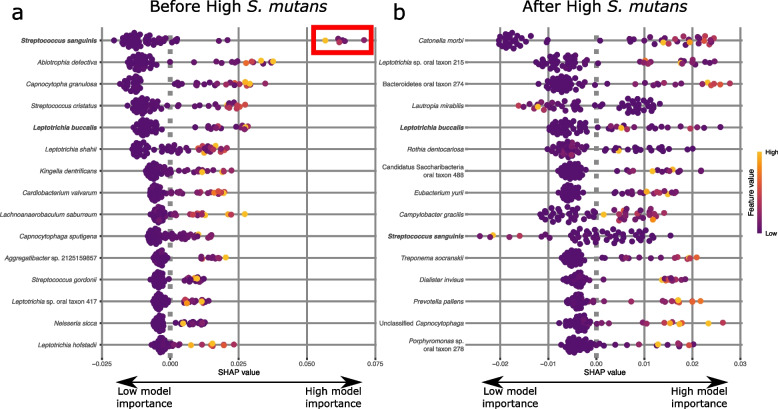
Taxa indicative of the community composition before and after high *S. mutans* relative abundance on a single tooth. Bee plots illustrate the importance of specific taxa in our random forest classification model to identify either (**a**) teeth before the proliferation of *S.* mutans or (**b**) teeth after the proliferation of *S. mutans*. SHAP value (SHapley Additive exPlanations) indicates the degree of importance for each variable on the model. Positive SHAP values indicate that that bacterial species is important for identifying the sample as belonging to the group in question while negative SHAP values indicate that the species is more important for the opposite group (i.e., before or after *S. mutans*). A SHAP value of zero indicates no impact on the model. For example, the red box in subpanel a is highlighting a subset of samples for which *Streptococcus sanguinis* is highly predictive of the sample being before the proliferation of *S. mutans*. Only the top ten taxa for either before or after high *S. mutans* relative abundance are shown. Color of points indicate how the feature value for that individual compared to the average for the entire population in which high feature value is indicated by lighter colored points. Bolded species names indicate species found in both Shapley plots

Evidence from cross-sectional studies (e.g., [31, 65]) suggest that caries disease progression is characterized by a rapid propagation of *S. mutans* and other acidogenic/aciduric bacteria during late-stage tooth decay, followed by a collapse of the community, and eventual recolonization. For our next analysis, we aimed to determine if this process is preceded or followed by predictable taxa in the plaque community. To better understand the temporal dynamics of the oral microbiome before and after high levels of *S. mutans*, we performed a random forest classification and post hoc explanatory analysis on individual teeth with a low relative abundance *S. mutans* (≤ 5%) either before or after the community on the same tooth had a high relative abundance of *S. mutans* (≥ 10%). Our random forest model had high classification accuracy for teeth with high *S. mutans* (during high *S. mutans* 83% correct) but had relatively low predictive accuracy for teeth designated as “before” or “after” high *S. mutans*. Accurate classification of teeth after high *S. mutans* was only 47% with most being misclassified as “during” and none as “before.” Teeth before high *S. mutans* were only classified correctly in 33% of cases with most being misidentified as during (50%) or after (17%). Taxa that were associated with teeth before high *S. mutans* include a variety of commensal species including *S. sanguinis*,* S. cristatus*,* S. gordonii*, *Abiotrophia defectiva*, *Aggregatibacter aphrophilus*, and* L. buccalis* as well as suspected opportunistic pathogens (e.g., *Leptotrichia shahii*, *Cardiobacterium valvarum*, *Kingella dentrificans*) (Fig. 3). Interestingly, the community after high *S. mutans* is distinct from that found before high *S. mutans* with the top explanatory taxa including *Cantonella morbi*, *Leptotrichia* sp. oral taxon 215, and *Bacteroidetes* oral taxon 274 (Fig. 3). Importantly, the lack of *S. sanguinis* is indicative of the community after colonization of high abundance of *S. mutans* which suggests that the community does not recover to its previous state, at least not initially or within the time period sampled here. More fine-grained longitudinal sampling is necessary to elucidate some of these patterns over time.

### HIV infection homogenizes the plaque microbiome across the posterior and anterior dentition

**Fig. 4:**
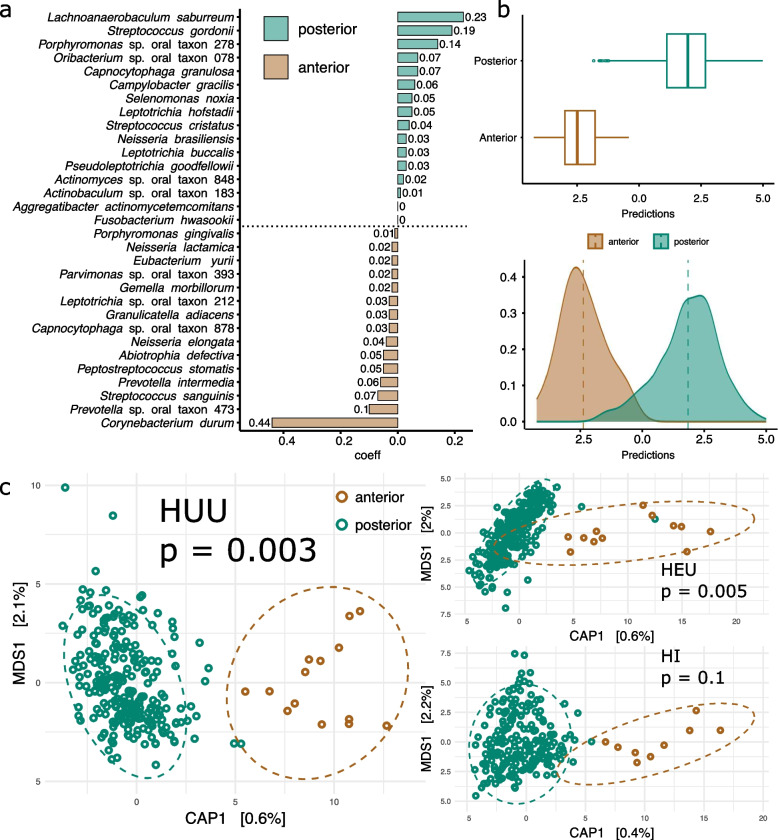
Community stratification of the anterior and posterior dentition is clear among HUU and HEU children but is dismantled in HI children. (a) Microbial signature of the anterior and posterior teeth among all individuals independent of HIV status. The relative weight of each taxon is listed as its coefficient value (*y* axis). (b) Prediction box and density plots of the microbial signature model for anterior and posterior teeth driven by the balance of taxa listed in subplot a. (c) Capscale plot depicting the Aitchison distance of anterior and posterior teeth in HUU, HEU, and HI children. Significance between groups determined by PERMANOVA analysis and listed as Bonferroni adjusted *p* values. Only permanent H-CF teeth were included in this analysis

Next, we investigated the impact of HIV status on the spatial distribution of the microbial community across the dentition of permanent teeth with no carious lesions (H-CF) from all three visits. We focused on healthy teeth only for this analysis to eliminate the effect of differences in oral health among the children. We detected conspicuous differentiation among the bacterial community colonizing the anterior dentition (i.e., central and lateral incisors, canines) as compared to the posterior dentition (i.e., premolars and molars) across all permanent teeth with the posterior teeth exhibiting a higher relative abundance of *Lachnoanaerobaculum saburreum*,* S. gordonii*, and *Porphyromonas* sp. oral taxon 278 and a more minor contribution of species belonging to the genera *Capnocytophaga*, *Campylobacter*, *Selenomonas*, *Leptotrichia*, *Streptococcus*, *Neisseria*, *Pseudoleptotrichia*, *Actinomyces*, *Actinobaculum*, *Aggregatibacter*, and *Fusobacterium*. Anterior teeth, conversely, were strongly associated with *C. durum* followed by *Prevotella* sp. oral taxon 473, and *S. sanguinis* followed by species belonging to the genera *Prevotella*, *Peptostreptococcus*, *Abiotrophia*, *Neisseria*, *Capnocytophaga*, *Granulicatella*, *Leptotrichia*, *Gemella*, *Parvimonas*, *Eubacterium*, and *Porphyromonas* (Fig. 4a, 4b). Importantly, however, this differentiation is primarily driven by HEU and HUU samples which exhibit a much stronger distinction between the anterior and posterior community composition as compared to children living with HIV. Statistical significance and effect size were assessed through a PERMANOVA analysis with 100 permutations for each HIV status group, where supragingival plaque samples collected from molars were randomly subsampled without replacement to the number of anterior teeth to control for sample size discrepancies. *p*-values were subsequently adjusted using FDR correction, and the average *p*-value was calculated across all 100 permutations. We found that the community distinction between anterior and posterior teeth in HUU (average FDR adjusted *p*-value = 0.003) and HEU samples (average FDR adjusted *p*-value = 0.005) is significant but not in children living with HIV (average FDR adjusted *p*-value = 0.143). However, the effect size for these observations is low (HUU *R*^2^ = 0.051, HEU *R*^2^ = 0.062, HI *R*^2^ = 0.064), reflective of the high variance in this dataset. Interestingly, using the same analysis described above, we found no significant differences between the communities inhabiting the mandibular and maxillary molars among any HIV status group (HI, average FDR *p*-value = 0.5, *R*^2^ = 0.03; HEU, average FDR *p*-value = 0.4, *R*^2^ = 0.03; HUU, average FDR *p*-value = 0.1, *R*^2^ = 0.03). Future studies should clarify these results as well as further consider the impact of buccal versus lingual orientated plaques on the oral microbiome.

### Depressed immune status is associated with a higher prevalence of cariogenic taxa

We next investigated the correlation of CD4 counts on the oral microbiome across all three visits. Across all samples, CD4 counts among HI children are significantly lower as compared to both HEU (*p* < 0.0001; Cohen’s *d* = − 0.33 ± − 0.44, − 0.22) and HUU children (*p* < 0.0001; Cohen’s *d* = − 0.40 ± − 0.51, − 0.29). Considering plaque samples collected at each visit, however, CD4 counts remained consistent among HI and HEU children, yet significantly decreased in visit three among HUU children as compared to both visit one (*p* = < 0.0001) and visit two (*p* = 0.00065) (Figure S8).

**Fig. 5:**
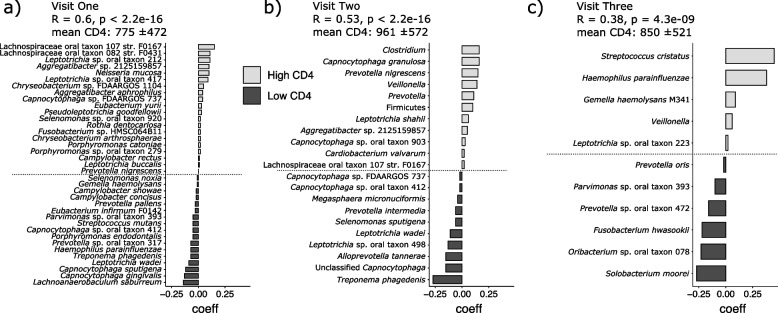
Balance of taxa associated with CD4 count among children living with HIV over all three clinical visits. **a** Balance of taxa at visit one that correspond to either high or low CD4 counts. **b** Balance of taxa at visit two that correspond to high or low CD4 counts. **c** Balance of taxa at visit three that correspond to high or low CD4 counts. The strength of the correlation between taxonomic groups is indicated by the R score and *p* value at each visit. Mean and standard deviation of the CD4 counts at each visit listed above each figure. Low CD4 count among children living with HIV is associated with diverse and discordant potential and actual cariogenic taxa, the signature of which is lessened with improving CD4 counts across the three visits

Next, we identified microbial signatures that were most predictive of CD4 counts in children living with HIV across our three sampling periods. We found that as CD4 counts increased over the three visits, the predictive power of microbial taxa decreased with visit one exhibiting the strongest correlation (*R* = 0.6, *p* < 2.2e-16) followed by visit two (*R* = 0.53, *p* < 2.2e-16) and the weakest correlation coefficient at visit three (*R* = 0.38, *p* = 4.3e-09) (Fig. 5). Taxa predictive of the lowest CD4 counts among children at visit one where the mean CD4 count is the lowest of our three sampling periods (775 ± 472) include a variety of taxa associated with the progression of caries disease including *S. mutans*, *Leptotrichia wadei*, and *L. saburreum* [86].

Conversely, high CD4 counts are associated with a variety of taxa previously identified as potentially protective against caries development (e.g., *Leptotrichia* sp. oral taxon 212 [87]) but also taxa that have been identified with higher caries risk (e.g., *Lachnospiraceae* bacterium oral taxon 082) [86]. At visit two where the mean CD4 count increased to 961 (± 572), fewer taxa were identified to be predictive of CD4 count but included some overlap between taxa identified in visit one including *L. wadei*, *Capnocytophaga* sp. oral taxon 412, *Lachnospiraceae* oral taxon 107 str. F0167, *C.* sp. FDAARGOS 737, and *Aggregatibacter* sp. 2,125,159,857.

While there are fewer pathogenic taxa contributing to the microbial signature of CD4 counts in visit two, *Selenomonas sputigena*, recently classified as a pathobiont capable of exacerbating the acidogenic activity of *S. mutans* in early childhood caries, is highly weighted in the correlation coefficients driving lower CD4 counts [86]. Finally, while the mean CD4 count among children living with HIV drops at visit three (850 ± 521), the strength of the association between the taxa identified as being microbial markers for high or low CD4 count, while statistically significant, is comparatively weak as compared to visits one and two (*R* = 0.38, *p* = 4.3e-09).

## Discussion

Our results suggest that the temporal and spatial dynamics of the supragingival plaque microbiome are altered by HIV infection and exposure. We hypothesize that these alterations are a consequence of a combination of factors including the following: (1) reduced salivary flow and increased salivary pH among children in this cohort, (2) differences in taxonomic plasticity and cohesion of the plaque community over time, and (3) immune-mediated alterations to the oral community. Moreover, our longitudinal approach bolsters previous observations [31, 65] that while *S. mutans* is a dominant member of the supragingival plaque community during caries intensification, it is not involved or does not play a major role in the initiation of tooth decay. Instead, our results suggest that within-species strain-level functional diversity may be a catalyst for community dysbiosis and tooth decay inception. Further strain-level functional analyses are required to elucidate the role of the supragingival plaque community in the initiation of tooth decay.

### Reduced salivary flow and increased salivary pH may be key contributors to alterations in the spatiotemporal structure of the supragingival plaque microbiome

Saliva provides several key benefits essential for maintaining homeostasis of the oral cavity including lubrication to facilitate swallowing and removal of food particles from the oral tissues, digestive enzymes including amylase for the digestion of carbohydrates into sugars, and antimicrobial compounds including hydrogen peroxide, lactoferrin, lysozymes, and a variety of antimicrobial peptides that modulate the colonization of oral tissues by microbes [88–90]. People living with HIV often experience chronic dry mouth (xerostomia), and children especially are affected by HIV-associated salivary gland disease (HIV-SGD) [91]. HIV-SGD affects the parotid, submandibular, and sublingual salivary glands and results in decreased saliva production and poorer quality saliva including decreased levels of sodium, calcium chloride, cystatin (essential for tooth remineralization), and lysozymes [91, 92]. While we could not empirically evaluate the impact of salivary flow and pH across all samples presented here, a small subset of individuals (*n* = 266) from this cohort were collected and analyzed for salivary pH and flow rate at the end of the sampling period (from January 2020 to February 2021). We propose that these measurements may reflect the broader patterns observed in this cohort. Specifically, comparisons of unstimulated salivary flow rates among children in this cohort found significant differences between HIV status groups. HUU children had the highest mean rate of salivary flow (0.33 mL/min) and HI children the lowest (0.22 mL/min) [93]. Importantly, the mean rate of salivary flow among HEU children was intermediate between HI and HUU at 0.27 mL/min [93], which may reflect long-lasting effects of the mother’s immune status on the oral biology of this growing cohort of children. Salivary gland hypofunction is associated with an increased risk of several oral diseases including caries disease and candidiasis [94–96]. Moreover, the mean pH of HI children in this cohort was significantly lower than HEU or HUU children which has important implications for the risk of tooth decay [93].

Given the biochemical properties of saliva and their importance in structuring the oral microbiome, we hypothesize that saliva is a primary contributor to differences in the biogeography of the supragingival plaque microbiome reported here. Location of individual teeth in the oral cavity, their proximity to major salivary ducts, morphological characteristics, and mechanical use (e.g., chewing vs tearing) influences their community composition. Previous research has found that the structure of the oral community follows an ecological gradient from the back to the front of the dentition so that the microbiome inhabiting the molars is distinct from that on the incisors [97–99]. This ecological pattern is clearly supported in the current study among HUU and HEU children. Conversely, individuals with impaired salivary flow due to injury or disease (e.g., Sjögren’s syndrome, see Proctor et al. [98]) experience a breakdown of this ecological gradient and a homogenization of the anterior and posterior oral communities, as is evident in our HI cohort. Interestingly, while the differences between the oral microbiome inhabiting anterior and posterior teeth in HEU children in the current study are significant, the distribution of these two communities is somewhat intermediate between HUU and HI children (see Fig. 4c), which may be reflective of their intermediate status in terms of salivary flow [93]. Previous studies have documented that disruptions in the natural ecological barriers that separate the human oral-pharyngeal and gut microbiome are symptomatic of disease [100, 101]. The results of this study suggest that these disruptions occur at much smaller spatial scales within the same host-associated habitat (i.e., the oral cavity) with possibly detrimental consequences towards overall oral and systemic health outcomes in HI children. Although we could not directly test these observations here, future studies on the temporal and spatial stability of the oral microbiome should incorporate the impact of salivary flow and composition, particularly among individuals with depressed immune status.

### Taxonomic turnover in the supragingival plaque microbiome may be adaptive response to changing host factors that is suppressed in disease

Using distance-based taxonomic turnover analyses, we found that the community inhabiting a single tooth may experience substantial taxonomic turnover over time, particularly among HUU children. This contrasts with expectations from previous longitudinal analyses of human-associated microbial ecosystems (primarily the gut microbiome) wherein large-scale shifts in the bacterial community are typically associated with illness or stress [57, 102, 103]. Given that bacteria inhabiting the oral cavity are subject to more direct and regular exchange with the external environment, it is possible that taxonomic turnover in the supragingival plaque microbiome is a signal of community health.

Specifically, we propose that temporal flexibility in the oral microbiome may reflect the community’s ability to rapidly adapt to environmental perturbations. Previous investigations into the temporal stability of the oral microbiome have suggested that it is more dynamic than other human-associated microbial habitats, with individual behavior and environment exerting a greater influence than genetic determinants on the oral community [13, 15, 19, 20]. However, other studies argue that the oral microbiome is relatively stable over time [104, 105]. These conflicting results may be attributed to differences in study design, such as the time scales measured, stability metrics used, and types of oral samples collected (e.g., saliva vs plaque), all of which could impact findings on microbial stability in the oral cavity. Our results suggest that community fluctuations over time may depend on both the individual’s health status and the specific temporal and spatial scales at which these observations are made. Importantly, we document a strong association between low taxonomic turnover and cariogenic taxa, including *S. mutans*, *P. multisaccharivorax*, and *P. denticola*. This association may indicate that diseased oral communities undergo less change over time due to lower baseline diversity and the dominance of acidogenic and aciduric taxa, potentially reflecting a suppressed adaptive capacity.

Importantly, while the participants in this study were asked to refrain from eating and oral hygiene practices before sample collection, we cannot exclude the possibility that some taxonomic turnover pattens observed here may result from differences in plaque maturation stages, with later stages generally being more diverse than initial tooth colonization. Future studies should assess plaque maturity with more rigorous methods to better evaluate its impact on stability analyses.

Despite substantial turnover on the individual tooth level, we do find clusters of taxa that are consistently co-associated over time within and across our HIV status groups. Thus, despite individualistic fluctuations in the overall community composition, a stable core of taxa is consistently present in the supragingival plaque microbiome, a pattern previously observed in longitudinal sampling of the oral cavity [15]. Clusters identified in the current study have similar functional expectations in that species that are associated with health or disease are consistently co-associated over time. For example, in our global core-association network, cluster 6 is made exclusively of known or suspected cariogenic taxa while cluster 1, cluster 2, cluster 5, and cluster 4 are composed primarily of oral symbionts and structural taxa. Importantly, while modularity (a measure of community cohesion over time) is roughly equivalent among HI, HEU, and HUU children, there are notable differences in the structure and composition of core-association networks among these three groups. The most conspicuous of these differences is the lack of cluster 6 and prominence of cluster 2 among HEU children. Given that cluster 6 is composed of cariogenic taxa, its absence in part may explain why, despite experiencing poor health outcomes in other areas that are similar to HI children [32–38], HEU children do not have a higher caries burden as compared to HUU children [28, 106, 107]. Importantly, however, cluster 2, which is prominent in HEU children as compared to HI and HUU children, contains taxa typically associated with periodontal disease. Due to the challenges in assessing pocket depth in children with mixed dentition and the focus of this project on caries disease, we were unable to conduct a comprehensive evaluation of periodontal health. However, gingival health was assessed using Oral Hygiene Index (OHI) assessments including gum bleeding on probing and plaque indices. It is thus unclear if these results suggest future or current periodontal disease risk among HEU children.

### Immune status among children living with HIV is associated with the abundance of cariogenic taxa

Individual immune status among children living with HIV has previously been associated with the prevalence and severity of caries disease (see, for example, [26]). In agreement with these findings, we find that low CD4 counts are strongly associated with the abundance of cariogenic taxa. While CD4 counts among children living with HIV appear to improve over the three time points collected here, given the impact of host immune status on the health of the oral cavity, we expect that continual monitoring of CD4 counts will be an important aspect of determining risk for tooth decay and other chronic health problems among HI children.

### Community dynamics before and after proliferation of *S. mutans*

Finally, while our random forest classification model was unable to precisely classify the oral community before and after high levels of *S. mutans*, we did detect specific taxa that were present before high *S. mutans* that were not retained after the recolonization of the tooth post *S. mutans* colonization. In particular, non-mutans streptococcal species including *S. cristatus*, *S. sanguinis*, and *S. gordonii* were indicative of the community before but not after *S. mutans* colonization and proliferation. Importantly, however, these patterns are driven by a small subset of samples which may be indicative of functional differences among strains of these non-mutans streptococci. For example, previous work has found substantial functional diversity among members of the streptococci, including *S. sanguinis* [108]. Moreover, while *S. sanguinis* is typically associated with oral health, high-resolution amplicon sequencing of *S. sanguinis* and other putatively commensal streptococci species has documented that some are strongly associated with later stages of caries disease [44]. Given that *S. sanguinis* is highly predictive of later *S. mutans* abundance among some of our samples, it may be the case that specific strains of *S. sanguinis* play a role in the initialization of caries while others are protective against caries [87]. Finally, our results suggest that the community inhabiting a tooth will not recover to its previous state after *S. mutans* is the dominant taxon. While these analyses are based on relative abundances (and therefore do not represent the actual load of *S. mutans* in each sample), monitoring these processes at smaller time scales may elucidate which taxa are predictive of the community before and after high levels of cariogenic taxa including *S. mutans*.

## Conclusions

The results of this study highlight the importance of scale—both temporally and spatially—in understanding the impact of the bacterial supragingival plaque community in the development of caries in the context of HIV infection and exposure. We find that HIV infection and exposure have a significant impact on the temporal and spatial structuring of the oral microbiome which may be the result of altered salivary flow and pH, individual host dynamics, and impaired immune status of individual children. Significantly, we find that children unexposed and therefore uninfected with HIV within our cohort exhibit relatively high taxonomic turnover of the supragingival plaque microbiome while maintaining cohesive and consistent groups of taxa over time. Additionally, we find that low taxonomic turnover is associated with higher frequencies of cariogenic taxa including *S. mutans*. Moreover, we find that HIV infection homogenizes the oral microbiome across the anterior and posterior dentition, with altered salivary function likely a key factor. A further appreciation of the temporal and spatial dynamics of the oral microbiome in health and disease may be necessary to identify molecular mechanisms of oral microbiome dysbiosis and the initiation, progression, and ultimate consequences of tooth decay among children living with and exposed to HIV.

## Supplementary Information

Supplementary Material 1.

Supplementary Material 2.

## Data Availability

The datasets generated and analyzed during the current study are available in the European Nucleotide Archive repository under accession number PRJEB76179. All preprocessing and analysis scripts are available at https://github.com/aemann01/long_oral_microbiome and are archived at Zenodo under the 10.5281/zenodo.11396312.

## Abbreviations

HUU: Child unexposed and therefore uninfected with HIV
HEU: Child exposed to but uninfected with HIV
HI: Child living with HIV
H-CF: A healthy tooth from a caries-free mouth
H-CE: A healthy tooth from a mouth with active enamel lesions
H-CD: Healthy tooth from a mouth with active dentin caries
E-CE: Tooth with an enamel lesion from a mouth with active enamel caries
E-CD: Tooth with an enamel lesion from a mouth with active dentin caries
D-CD: Tooth with a dentin cavity from a mouth with active dentin cavities
