# Supplementary material for: HIV infection and exposure is associated with increased cariogenic taxa, reduced taxonomic turnover, and homogenized spatial differentiation for the supragingival microbiome
Source: Microbiome. 2025 Jun 16;13:144. doi: 10.1186/s40168-025-02123-9 (PMC12168284; doi:10.1186/s40168-025-02123-9)
Supplement: Supplementary file 2 — Supplementary Material 1. [file 40168_2025_2123_MOESM1_ESM.docx]

Exploring the effects of HIV infection and exposure on cariogenic taxa, taxonomic turnover, and spatial differentiation in the supragingival plaque microbiome

Allison E. Mann, Ciara Aumend, Suzanne Crull, Lauren M. O’Connell, Esosa Osagie, Paul Akhigbe, Ozoemene Obuekwe, Augustine Omoigberale, Matthew Rowe, Thomas Blouin, Ashlyn Soule, Colton Kelly, the DOMHaIN Study Team, Robert A. Burne, Modupe O. Coker, Vincent P. Richards

**Supplementary Figures**

**Figure S1: Rarefaction analysis of all samples.** Samples are split by oral health group. Line colors indicate HIV health group of individual.


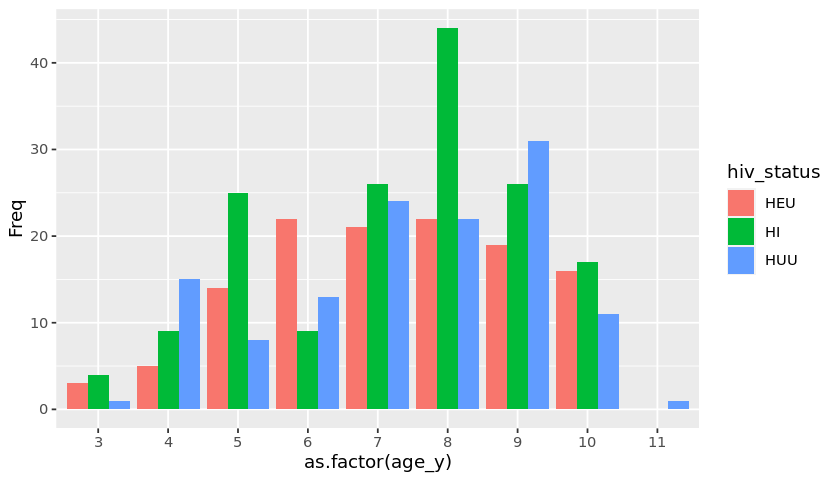


**Figure S2: Age distribution across all individuals by HIV status group**


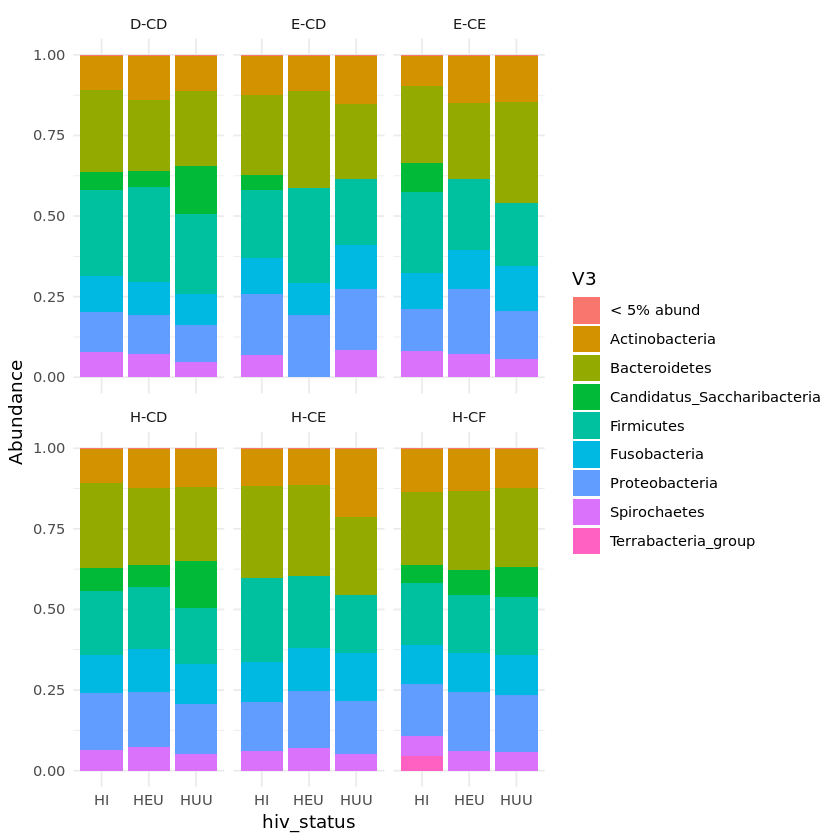


**Figure S3: Relative abundance of major phyla found in each HIV status and tooth health group.** Phyla with an average relative abundance of less than 5% of the dataset are collapsed to improve readability.


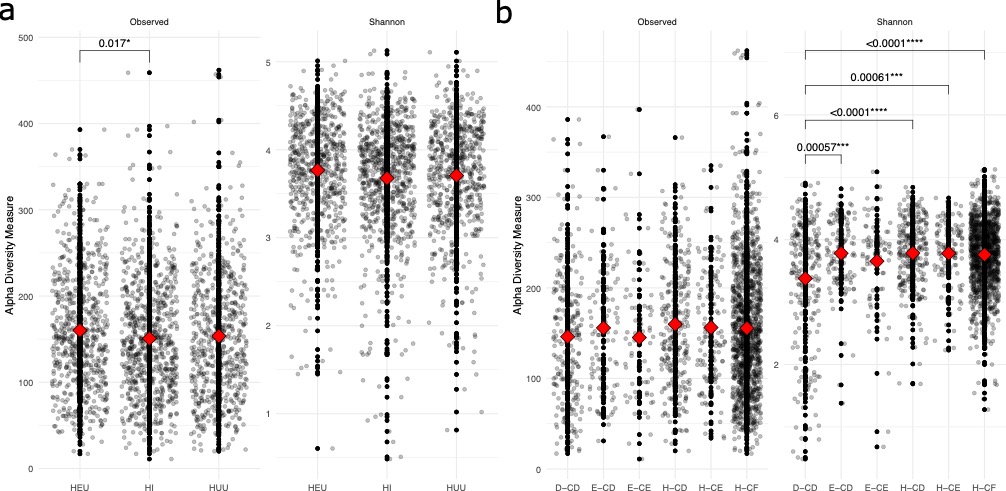


**Figure S4: Alpha diversity.** Shannon diversity and the number of observed ASV by (a) HIV status group and (b) individual tooth health category.

**Figure S5: Maximum likelihood tree of full-length *Treponema* spp. *rpo*C genes and evolutionary placement of putative unknown *Treponema* lineage detected in this study.** Reference tree includes all *Treponema* species in our custom *rpo*C database. Red star indicates likely relationship of a single ASV previously assigned to *Treponema phagedenis* using an evolutionary placement algorithm. The ASV is distinct from *T. phagedenis* and sister to oral *Treponema* species. However, given its placement in the tree, it is likely that it represents an unknown *Treponema* species that does not exist in our database.

**Figure S6: Correlation between age and predictive taxa using balance of taxa analysis.** Y axis is age of child at time of sampling.

**Figure S7: Correlation between sex and predictive taxa using a balance of taxa analysis.** Positive predictions are more highly associated with males (purple) as opposed to females (orange). Dotted lines indicate mean prediction coefficient for each group.

**Figure S8: CD4 counts fluctuate over time in HUU children but not in HI or HEU children.** Jitter plot of individual CD4 counts (by plaque sample) over each sampling visit for each of the three HIV status groups. Dotted lines represent the typical range of CD4 counts in healthy individuals (500 to 1500 cells/mm^3^). Red diamond represents median value for each group.
